# Biokinetic Characterization and Activities of N_2_O-Reducing Bacteria in Response to Various Oxygen Levels

**DOI:** 10.3389/fmicb.2018.00697

**Published:** 2018-04-10

**Authors:** Toshikazu Suenaga, Shohei Riya, Masaaki Hosomi, Akihiko Terada

**Affiliations:** Department of Chemical Engineering, Tokyo University of Agriculture and Technology, Koganei, Japan

**Keywords:** nitrous oxide reduction, O_2_ inhibition, heterotrophic denitrification, biokinetic analysis, microsensor

## Abstract

Nitrous oxide (N_2_O)-reducing bacteria, which reduce N_2_O to nitrogen in the absence of oxygen, are phylogenetically spread throughout various taxa and have a potential role as N_2_O sinks in the environment. However, research on their physiological traits has been limited. In particular, their activities under microaerophilic and aerobic conditions, which severely inhibit N_2_O reduction, remain poorly understood. We used an O_2_ and N_2_O micro-respirometric system to compare the N_2_O reduction kinetics of four strains, i.e., two strains of an *Azospira* sp., harboring clade II type *nosZ*, and *Pseudomonas stutzeri* and *Paracoccus denitrificans*, harboring clade I type *nosZ*, in the presence and absence of oxygen. In the absence of oxygen, the highest N_2_O-reducing activity, *V*_m,N2O_, was 5.80 ± 1.78 × 10^−3^ pmol/h/cell of *Azospira* sp. I13, and the highest and lowest half-saturation constants were 34.8 ± 10.2 μM for *Pa. denitirificans* and 0.866 ± 0.29 μM for *Azospira* sp. I09. Only *Azospira* sp. I09 showed N_2_O-reducing activity under microaerophilic conditions at oxygen concentrations below 110 μM, although the activity was low (10% of *V*_m,N2O_). This trait is represented by the higher O_2_ inhibition coefficient than those of the other strains. The activation rates of N_2_O reductase, which describe the resilience of the N_2_O reduction activity after O_2_ exposure, differ for the two strains of *Azospira* sp. (0.319 ± 0.028 h^−1^ for strain I09 and 0.397 ± 0.064 h^−1^ for strain I13) and *Ps. stutzeri* (0.200 ± 0.013 h^−1^), suggesting that *Azospira* sp. has a potential for rapid recovery of N_2_O reduction and tolerance against O_2_ inhibition. These physiological characteristics of *Azospira* sp. can be of promise for mitigation of N_2_O emission in industrial applications.

## Introduction

Nitrous oxide (N_2_O) is an ozone-depleting and greenhouse gas (Ravishankara et al., 2009), therefore it is important to decrease N_2_O emissions from natural ecosystems, agriculture, and industrial systems. A large fraction of N_2_O is emitted from agricultural croplands (IPCC, 2013; Harter et al., 2014). Emissions from industrial systems, mainly wastewater treatment plants, have become more significant as a result of upgrading of biological nitrogen removal processes, i.e., nitrification–denitrification or partial nitrification–anammox processes (Law et al., 2012). N_2_O is produced via multiple biological and abiotic pathways, e.g., in denitrification as an intermediate (Philippot et al., 2011; Wunderlin et al., 2011; Ishii et al., 2014), in the nitrifier denitrification of ammonia-oxidizing microorganisms (Zhu et al., 2013; Ali et al., 2016), and in chemical oxidation of hydroxylamine (Soler-Jofra et al., 2016; Terada et al., 2017). It is consumed mainly by denitrifying bacteria harboring a N_2_O reductase system (Nos) (Henry et al., 2006; Zumft and Kroneck, 2006; Jones et al., 2008; Pauleta et al., 2013). In subsequent denitrification steps, N_2_O reduction is severely affected by environmental factors, i.e., pH, availability of electron donors, and dissolved oxygen (DO) (Law et al., 2012; Pan et al., 2013). Although the physiological traits on N_2_O reduction by canonical denitrifying species, i.e., the genera *Pseudomonas* and *Paracoccus* (Zumft, 1997; Vollack and Zumft, 2001; Philippot, 2002; Read-Daily et al., 2016), have been studied to date, knowledge is still limited. Comprehensive and thorough physiological research on N_2_O-reducing bacteria under various environmental conditions is therefore warranted.

Recent metagenomic analyses have shown that N_2_O-reducing bacteria that harbor Nos can be classified into two clade types: clade I and clade II (Sanford et al., 2012; Jones et al., 2013). It has been reported that the abundances of the two clades potentially depend on environmental conditions, e.g., pH, concentration of calcium ion, and C/N ratio, and niche differentiation probably occurs because of their physiological characteristics (Jones et al., 2014; Domeignoz-Horta et al., 2015; Juhanson et al., 2017). Some clade II type N_2_O-reducing bacteria reportedly have lower half-saturation constants for N_2_O than do those affiliated to clade I type bacteria, suggesting that a low N_2_O concentration favors growth of clade II type N_2_O-reducing bacteria (Yoon et al., 2016). Additionally, most non-denitrifying N_2_O-reducing bacteria, which are unable to reduce nitrite and nitrate, are clade II type (Sanford et al., 2012; Hallin et al., 2018). Given these traits of clade II type bacteria, reports suggest that they potentially play an important role as N_2_O sinks (Jones et al., 2014; Orellana et al., 2014).

Studies of the oxygen effects are of fundamental and engineering importance. The DO concentration determines N_2_O emissions from soils and wastewater treatment plants via mainly nitrifier–denitrification and heterotrophic denitrification (Tallec et al., 2006; Morley et al., 2008; Riya et al., 2012). Studies on gene transcription and enzyme expression have suggested that Nos expression is regulated by the DO concentration (Körner and Zumft, 1989; Bergaust et al., 2012) and, more importantly, that the Nos enzyme is inactivated by oxygen (Pauleta et al., 2013). In addition, the susceptibility of N_2_O-reducing activity to the oxygen concentration is distinct at species or strain levels, as reported for the genera *Thauera* and *Pseudomonas* (Miyahara et al., 2010; Liu et al., 2013; Zheng et al., 2014). However, the influence of O_2_ on N_2_O reduction by clade II type N_2_O-reducing bacteria has not been comprehensively studied, except *Gemmatimonas aurantiaca* strain T-27 (Park et al., 2017). To enable their use in engineering applications as N_2_O sinks, the effects of oxygen on the N_2_O reduction activities of clade I and II type N_2_O-reducing bacteria need to be compared based on biokinetic analysis.

In this study, we, for the first time, compared the N_2_O reduction kinetics of clade I and clade II type N_2_O-reducing bacteria in the presence and absence of oxygen. *Pseudomonas stutzeri* and *Paracoccus denitrificans*, harboring *nosZ* clade I type, and two strains of *Azospira* sp., isolated from a N_2_O-fed enrichment device (unpublished data) inoculated with municipal wastewater treatment biomass, were subject to biokinetic comparison.

## Materials and methods

### Bacterial strains and culture conditions

Two *Azospira* sp. strains and two strains of canonical denitrifiers, i.e., *Ps. stuzeri* strain JCM5965 (ATCC17588) and *Pa. denitrificans* strain NBRC102528 (ATCC17741), were used in this study. *Azospira* sp. strains I09 and I13, classified as Betaproteobacteria, were isolated from enrichment devices supplying N_2_O as an electron acceptor (unpublished data). *Ps. stutzeri* and *Pa. denitrificans* were chosen because they are widely used as canonical denitrifiers (Körner et al., 1987; Baumann et al., 1996; Miyahara et al., 2010; Black et al., 2016). For comparison with other biokinetic studies, weight of a bacterial cell was determined as 0.388, 0.604, 0.743, and 0.787 pg-dry weight/cell for *Azospira sp*. strain I09, I13, *Ps. stutzeri* and *Pa. denitrificans*, respectively.

All the strains were aerobically pre-grown in an autoclaved nutrient medium containing (per liter of distilled water) 5.0 g of Bacto Peptone (BD-Difco, NJ), 3.0 g of Oxoid™ Lab-Lemco meat extract (Thermo Scientific, MA), and 5.0 g of NaCl.

### Chemical analyses

Dissolved organic carbon and dissolved total nitrogen were determined with a TOC analyzer, installing a total nitrogen measurement unit (5000A, Shimadzu, Kyoto, Japan). pH was measured using a pH meter (F-52, HORIBA, Kyoto, Japan). The gaseous N_2_O concentration was determined using a gas chromatograph with an electron-capture detector (GC-14B, Shimadzu, Kyoto, Japan) instrument. The measurement conditions were described in a previous paper (Terada et al., 2013).

### Activity measurements

The pre-grown bacterial strains were harvested in centrifuge tubes at the early stationary phase. Two strains of *Azospira* spp. (I09 and I13) were washed twice with 0.05 × phosphate-buffered saline (PBS) by centrifugation at 5,000 rpm for 5 min, and re-suspended in the experimental medium. Our preliminary experiment showed that the N_2_O-reducing activities of *Ps. stutzeri* JCM5965 and *Pa. denitrificans* NBRC102528 were significantly reduced by washing by centrifugation (data not shown). The pre-incubated cell suspensions of these two strains were therefore diluted with the experimental medium instead of washing. The experimental medium was placed in a conical flask, and the flask was sealed with a silicone cap (Shin-Etsu Polymer Co., Tokyo, Japan). The medium was mixed by shaking at 100 rpm at 30°C. The phosphate buffer medium, devoid of electron donors, contained (per liter of distilled water) 100 mg of KH_2_PO_4_, 6.6 mg of NaCl, 8.20 mg of MgSO_4_·7H_2_O, 13.4 mg of KCl, 115 mg of NH_4_Cl, 188 mg of NaHCO_3_, and 1 mL of a trace element solution consisting of (per liter of distilled water) 10 g of FeSO_4_ 7H_2_O, 10 g of FeCl_3_ 7H_2_O, 2 g of ZnSO_4_ 7H_2_O, 4 g of CuSO_4_ 7H_2_O, 0.5 g of NaMoO_4_ 2H_2_O, 0.1 g of MnCl_2_ 4H_2_O, 0.1 g of H_3_BO_4_, 0.3 g of Na_2_SeO_3_, and 10 g of citric acid (Miyahara et al., 2010). After sterilization, pH of the medium was adjusted to 7.5 with 1 M HCl.

**Figure 1:**
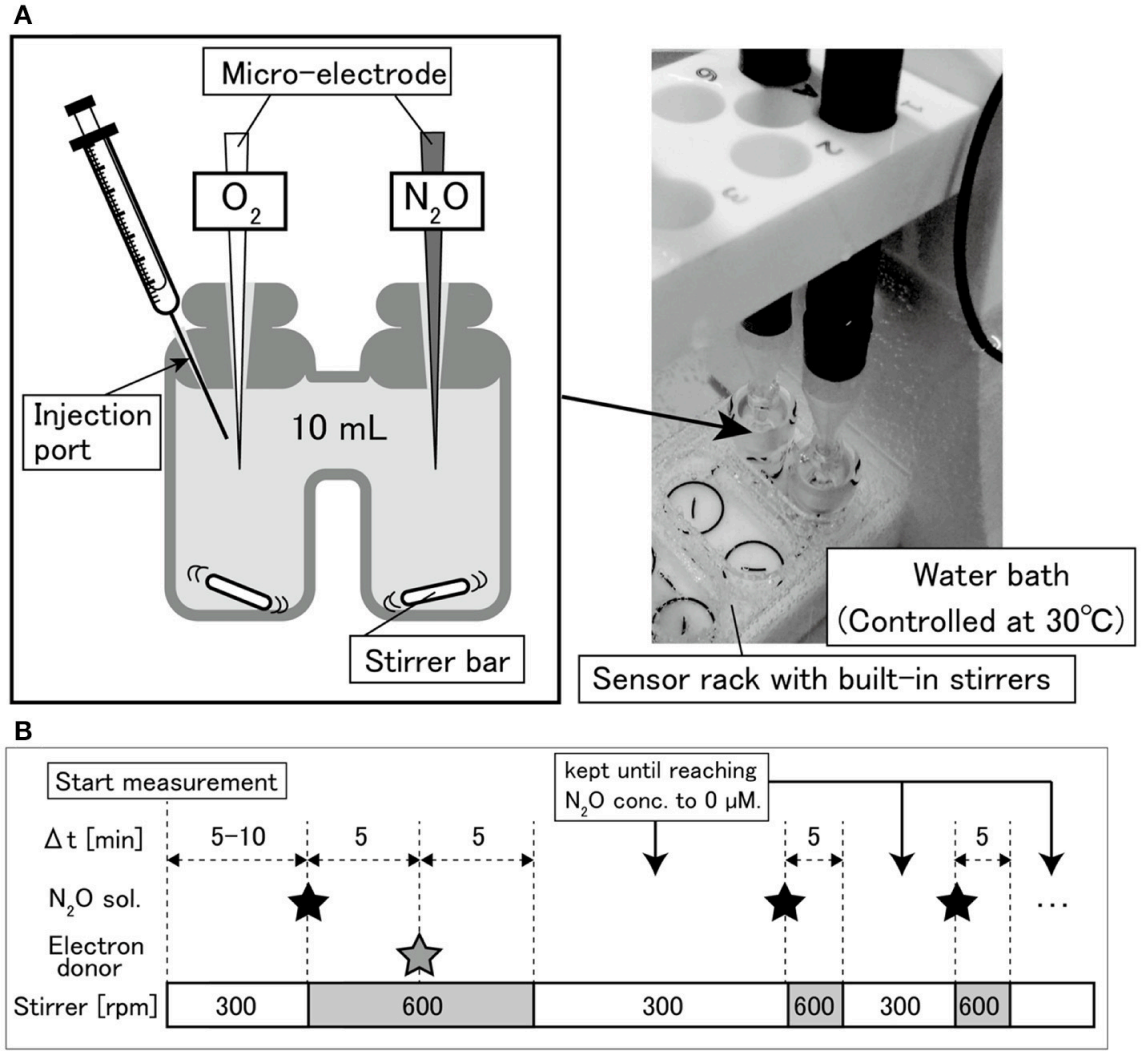
**(A)** Diagram of double-port chamber and **(B)** experimental sequence of N_2_O and O_2_ respiration measurements.

The O_2_ and N_2_O consumption behaviors were investigated using an O_2_ and N_2_O micro-respiration system with amperometric microsensors (Unisense, Aarhus, Denmark). This system consisted of a Clark-type N_2_O sensor (N2O-MR), O_2_ sensor (OX-MR), double-port chamber (10 mL, MR-Ch double port), and stirrers installed in a sensor stand (Figure 1). The temperature during the experiments was controlled at 30 ± 0.2°C by a water bath. The experimental sequences of O_2_ and N_2_O consumption are shown in Figure 1B. The chamber was filled with the cell suspension, in which electrodes were inserted. Highly concentrated N_2_O water (27.05–24.09 mM at 20–25°C and 0% salinity Weiss and Price, 1980) was prepared by pouring 25 mL of deionized water in a 50 mL vial, supplying pure N_2_O gas to the water for 10 min at a room temperature and subsequently sealing the vial with a butyl rubber stopper. The highly concentrated N_2_O water with a volume of 15–20 μL was injected into the chamber from the injection port (Figure 1A) using a Hamilton syringe, to replace the bacterial cell suspension and to give an initial dissolved N_2_O concentration of approximately 50 μM. Then 250 mM sodium acetate solution (25 μL) was added to adjust the initial concentration to 625 μM. To achieve a homogeneous condition immediately, the glass-coated stir bar, was stirred at 600 rpm for 5 min after N_2_O and carbon source injection and then at 300 rpm during the measurements. O_2_ and N_2_O concentrations were continuously monitored using SensorTrace Suite ver.2.8.0 (Unisense, Aarhus, Denmark). After the N_2_O was completely consumed, highly concentrated N_2_O water was injected again. The number of N_2_O injections was changed depending on the distinct trends in the N_2_O reduction rates observed among the tested strains. After the measurements, a sample of the cell suspension was immediately taken from the chamber and fixed with a 2% glutaraldehyde solution to determine the cell number, as previously described (Lunau et al., 2005). Briefly, the cell-counting procedure was as follows. Ethanol was added to the fixed cell suspension to achieve a final concentration of 10%. The mixture was homogenized for 10 s at 10 W (VP-050, Taitec, Tokyo, Japan), followed by dilution with 0.05 × PBS. The cells were trapped on a 0.2 μm membrane filter (Isopore, Merck Millipore, Germany), and washed twice with 1 mL of sterilized Tris-acetate EDTA buffer. The cells on the filter were stained with Moviol-SYBR Green I (Thermo Fisher Scientific, MA) and enumerated under a fluorescence microscope (BZ-8100, Keyence, Osaka, Japan).

### Kinetic parameter estimation

The O_2_ and N_2_O concentration profiles were smoothed with the function of Sigma Plot 13.0 (Systat software, CA) to remove high-frequency noise and instantaneous reduction rate (Δ = 3–5 s) was calculated and normalized with cell number (*V*s [pmol/h/cell]). The maximum O_2_ and N_2_O uptake rates (*V*_m,O2_ and *V*_m,N2O_ [pmol/h/cell]) and the half-saturation constants for O_2_ and N_2_O (*K*_m,O2_ and *K*_m,N2O_ [μM]) were determined by fitting the profiles to the Michaelis–Menten equation (Equation 1) (Martens-Habbena et al., 2009). The *V*_m,N2O_ value was calculated from the last measurement of the N_2_O profiles.

(1) $$V_{s}=\frac{V_{\text{m},\text{S}}\cdot S}{K_{\text{m},\text{S}}+S}$$

where *S* [μM] is the concentration of either O_2_ or N_2_O. To compare the N_2_O reduction activities among the strains, the specific affinity for N_2_O (*a*_0,N2O_ [L/cell/h]) was calculated as follows (Equation 2):

(2) $$a_{\text{0},\text{N2O}}=\frac{V_{\text{m},\text{N2O}}}{K_{\text{m},\text{N2O}}}\times 10^{-6}$$

Statistical analysis was performed with ANOVA (Tukey HSD) in SPSS Statistics (IBM, NY), and statistical significance was evaluated by *p*-value below 0.05 as a threshold.

The effect of O_2_ on the N_2_O-reducing activity was investigated by fitting the experimental profiles to a mathematical model. The heterotrophic denitrification model, incorporating the O_2_ inhibition (Ni et al., 2011), was used to estimate the degree of O_2_ inhibition to N_2_O uptake rates. The terms of an electron donor, nitric oxide (NO) and ammonium (${\text{NH}}_{4}^{+}$) were excluded in the model proposed by Ni et al. (2011) (Equation 3) because theoretically NO is not produced and organic carbon and ${\text{NH}}_{4}^{+}$ were in excess in the medium (i.e., addition of ${\text{NH}}_{4}^{+}$, organic carbon, and N_2_O under anoxic conditions).

(3) $$\frac{dS_{\text{N2O}}}{dt}=V_{\text{m},\text{N2O}}\frac{S_{\text{N2O}}}{K_{\text{m},\text{N2O}}+S_{\text{N2O}}}\frac{K_{\text{I},\text{O2}}}{K_{\text{I},\text{O2}}+S_{\text{O2}}}X\times 10^{-6}$$

where *X* [cells/L] is the concentration of bacterial cells in the chamber, and *K*_I,O2_ [μM] is the O_2_ inhibition coefficient. For *Azospira* sp. strains I09, I13, and *Ps. stutzeri, K*_I,O2_ values were estimated by fitting the 1st-spiked N_2_O profile to the model based on the least-squares method using the solver function of Microsoft Excel ver. 15.26. For *Pa. denitrificans*, determination of *K*_I_,_O2_ was not feasible because the trend of N_2_O consumption is not explainable by the model. The lowest *K*_I,O2_ value detected in this study was 0.1 μM due to the detection limit of an O_2_ microelectrode.

The relative activity of N_2_O reduction rate *E* [dimensionless] under an anoxic condition was calculated by Equation (4).

(4) $$E=\frac{V_{\text{N2O}}}{V_{\text{m},\text{N2O}}}$$

*V*_N2O_ was attained from an N_2_O profile with the concentration range from 10 to 40 μM under an anoxic condition. The Nos activation rate (*V*_Nos_ [h^−1^]) was defined as a degree of activity recovery of N_2_O reduction after changing an aerobic to anoxic condition. *V*_Nos_ value was acquired by linear approximation of *E* as a function of elapsed time after DO concentration becomes zero (*t*_anoxic_). The analysis was performed with Sigma Plot 13.0.

### Quantifying transcripts of *nosZ* gene

Dynamics of *nosZ* gene transcripts of *Azospira* sp. strain I09 were quantified by reverse-transcription quantitative PCR (RT-qPCR). A medium, adding (per liter of distilled water) 0.20 g of sodium acetate and 0.050 g of NH_4_Cl to the synthetic medium for the respirometric test, was autoclaved, followed by pH adjustment at 7.5 with 1 M HCl. *Azospira* sp. strain I09 was inoculated and aerobically pre-grown at 30°C in a 500 mL bottle until the late-exponential growth phase (OD600 = 0.121 after incubation for 23 h). After the aerobic pre-growth, different gases were supplied via a sterilized filter (HEPA-VENT, GE Healthcare, UK) at three different phases: 1 L/min of air in Phase 1, 0.5 L/min of N_2_ in Phase 2, and 0.5 L/min of 100 ppm N_2_O/N_2_ in Phase 3. The gas flow rate was controlled using a mass flow controller (HORIBA, Kyoto, Japan). DO concentration in the medium was monitored using a DO meter (FDO Multi3410, WTW, Weilheim in Oberbayern, Germany). At each sampling point, cell suspension (10 mL) was transferred to a 15 mL tube and centrifuged at 10,000 rpm for 5 min at 4°C, and the supernatant was subsequently decanted. One milliliter of RNApro solution (FastRNA Pro Blue Kit, MP Biomedicals, CA) as a retardant of RNA degradation was immediately added to completely re-suspend the pellet according to the manufacture's protocol, followed by storage at 4°C until RNA extraction. RNA was extracted with a FastRNA Pro Blue Kit (MP Biomedicals, CA) and quantified by a UV-Vis spectrophotometer (Nanodrop 2000c, Thermo Fisher Scientific, MA). The extracted RNA was reverse-transcribed to complementary DNA with a QuantiTect Reverse Transcription Kit (QIAGEN, Hilden, Germany). The clade II type *nosZ* and 16S rRNA genes were quantified by real-time qPCR using a CFX96 Real-Time PCR Detection System (BioRad Laboratories, CA). The primer sets for clade II type *nosZ* and 16S rRNA genes were nosZ-II-F (5′-CTIGGICCIYTKCAYAC-3′)—nosZ-II-R (5′-GCIGARCARAAITCBGTRC-3′) (Jones et al., 2013) and 341f (5′-CCTACGGGAGGCAGCAG-3′)−517r (5′-ATTACCGCGGCTGCTGG-3′) (Muyzer et al., 1993), respectively. PCR for 16S rRNA gene amplification was initiated with initial denaturation at 95°C for 2 min, followed by 40 cycles at 95°C for 30 s, 60°C for 30 s, and 72°C for 30 s. PCR for the clade II type *nosZ* gene was initiated with denaturation at 95°C for 1 min, followed by 50 cycles at 95°C for 15 s, 54°C for 30 s, 72°C for 30 s, and 80°C for 30 s. The reaction buffer for the 16S rRNA gene consisted of 10 μL of SsoFast™ EvaGreen® Supermix (BioRad Laboratories, Hercules, CA), 1 μL of 10 mM forward and reverse primers, 5 μL of template, and 3 μL of distilled water. The PCR buffer for the clade II type *nosZ* gene consisted of 10 μL of SYBR Premix Ex Taq II (Tli RNaseH Plus, Takara Bio, Shiga, Japan), 2 μL of 10 mM forward and reverse primers, 1 μL of distilled water, and 5 μL of template. Plasmid DNA (pGEM-T Easy Vector, Promega, WI) containing each gene was transformed into *Escherichia coli* competent cells (Competent High DH5α, Toyobo, Osaka, Japan) and the plasmids were isolated for each standard solution with a plasmid extraction kit (MagExtractor -Plasmid-, Toyobo, Osaka, Japan). After confirmation of insertion of each functional gene by Sp6 and T7 primers (Huang et al., 2005), the plasmid DNA as a positive control was diluted to obtain standard solutions containing 1.0 × 10^8^ to 1.0 × 10^2^ copies per 5 μL in series. To increase the PCR efficiency, the plasmid containing the *nosZ* clade II amplicon was linearized with the restriction enzyme *Eco*RI (Takara bio, Kyoto, Japan) and used as a standard solution. The gene transcripts of *nosZ* were normalized with the amount of total RNA and gene transcripts of 16S rRNA to trace dynamics of *nosZ* gene expression. The transcripts of 16S rRNA gene was in the same order of magnitude during the tested three phases (Data not shown).

## Results

### Activity measurements and biokinetic comparison

**Figure 2:**
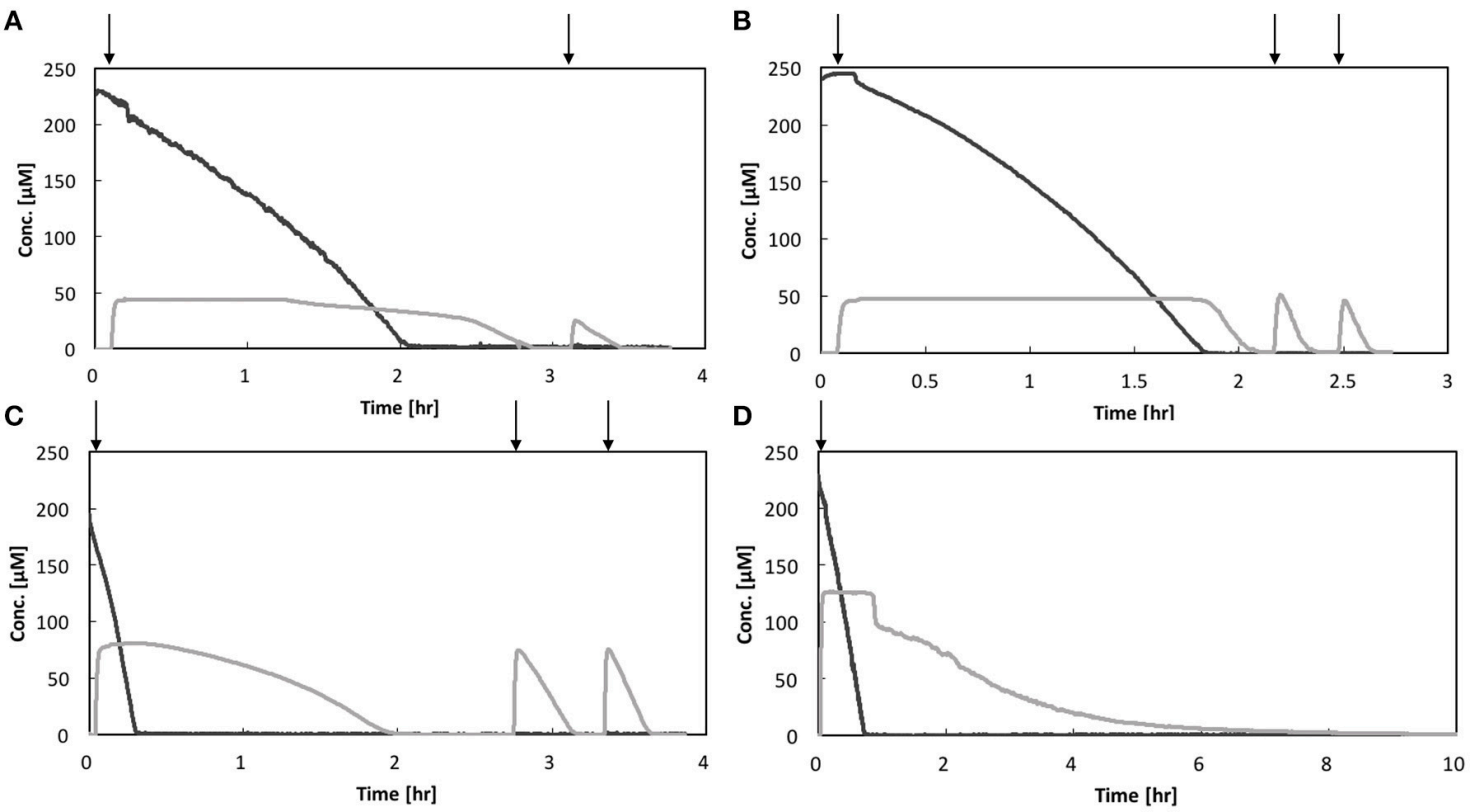
N_2_O and O_2_ respiration profiles of isolates: **(A)** *Azospira* sp. strain I09, **(B)** *Azospira* sp. strain I13, **(C)** *Pseudomonas stutzeri* JCM5965, and **(D)** *Paracoccus denitrificans* strain NBRC102528. Black line: O_2_, gray line: N_2_O. Vertical arrows indicate N_2_O spikes.

Activity measurement of each strain was conducted in triplicate. The representative O_2_ and N_2_O concentrations profiles are shown in Figure 2 and the two other replicates in SI (Figures S1–S4). All the bacterial strains showed facultative N_2_O-reducing activities, consuming O_2_ prior to N_2_O. However, the O_2_ concentration at which N_2_O-reducing activity was initiated was not consistent for each strain. Except in the case of *Pa. denitrificans*, the first-spiked N_2_O was completely consumed, followed by injection of N_2_O-concentrated liquid. For *Azospira* sp. strains I09 and I13, the maximum N_2_O reduction rate reached a plateau at the second or third additional N_2_O injection. The maximum N_2_O reduction rate of *Azospira* sp. I09 after the second injection was normalized to 114 ± 8% (*n* = 3) higher than that after the first injection. The analogous trend was attained for *Azospira* sp. I13, displaying that the maximum N_2_O reduction rates after the second and third injections were 132 ± 14% (*n* = 3) and 132 ± 8% (*n* = 2) of the rate after the first one, respectively. For *Ps. stutzeri*, the maximum N_2_O reduction rates after the additional injections further increased: 224 ± 69% (*n* = 3) for the second, 267 ± 62% (*n* = 3) for the third, 280 ± 40% (*n* = 2) for the fourth, and 294 ± 29% (*n* = 2) for the fifth, higher than the rate after the first injection. *Pa. denitrificans* was spiked with N_2_O once because the reduction rate slowed down below 50 μM N_2_O, entailing 10 h to consume the first-spiked N_2_O (Figure S4). The initial N_2_O concentration hampered accurate measurement for the N_2_O reduction activity of *Pa. denitrificans*; therefore, the volume of injected N_2_O solution was adjusted to ensure a higher N_2_O concentration (100–150 μM) (Figure 2D and Figure S4). The maximum N_2_O uptake rate (*V*_m,N2O_) and half saturation constant for N_2_O (*K*_m,N2O_) were estimated by fitting the Michaelis–Menten equation to the N_2_O profile at the final injection in each run (Table 1). *Azospira* sp. I13 showed the highest *V*_m,N2O_, i.e., 5.80 ± 1.78 × 10^−3^ pmol/h/cell, among all the strains, followed by *Ps. stutzeri* (1.64 ± 0.34 × 10^−3^ pmol/h/cell), *Azospira* sp. I09 (6.34 ± 0.8 × 10^−4^ pmol/h/cell), and *Pa. denitrificans* (5.01 ± 1.0 × 10^−4^ pmol/h/cell). The highest and lowest *K*_m,N2O_ values were 34.8 ± 10.2 μM for *Pa. denitirificans* and 0.866 ± 0.29 μM for *Azospira* sp. I09, respectively. The other two strains displayed comparable *K*_m,N2O_ values of about 4 μM. The N_2_O affinities (*a*_0,N2O_) of *Azospira* sp. strains I13 and I09 were higher than those of *Ps. stutzeri* and *Pa. denitirificans* because of the inherently higher *V*_m,N2O_ (I13) or lower *K*_m,N2O_ (I09).

**Table 1:** Biokinetic parameters for the tested strains.

**Strains**	***nosZ* type**	***V*_m,O2_ [ × 10^−3^ pmol/h/cell]**	***K*_m,O2_ [μM]**	***a*_0, O2_ [ × 10^−9^ L/cell/h]**	***V*_m,N2O_ [ × 10^−3^ pmol/h/cell]**	***K*_m,N2O_ [μM]**	***a*_0, N2O_ [ × 10^−9^ L/cell/h]**	***V*_Nos_ [h^−1^]**	***K*_I, O2_ [μM]**
*Azospira* sp. I09	II	1.13 (0.43)^a^	2.90 (1.69)^a^	0.391	0.634 (0.08)^a^	0.866 (0.29)^a^	0.732	0.319 (0.028)	2.33 (1.7)
*Azospira* sp. I13	II	3.70 (0.45)^b^	1.68 (0.33)^a^	2.21	5.80 (1.78)^b^	3.76 (1.99)^a^	1.54	0.397 (0.064)	0.330 (0.204)
*Ps. stutzeri* JCM5965	I	3.03 (0.60)^b^	5.94 (1.05)^a^	0.510	1.64 (0.34)^a^	4.01 (0.77)^a^	0.408	0.200 (0.013)	0.164 (0.042)
*Pa. denitrificans* NBRC102528	I	4.07 (1.11)^b^	5.39 (3.21)^a^	0.756	0.501 (0.10)^a^	34.8 (10.2)^b^	0.0144	*n.a*.^**^	*n.a*.^**^

*These values were acquired from N_2_O activity measurements in triplicate. Values in parentheses represent standard deviations*.

^*^Statistically different values (p < 0.05) are distinguished by superscripts a and b.

** *Not applicable*.

### Effects of O_2_ on N_2_O reduction

**Figure 3:**
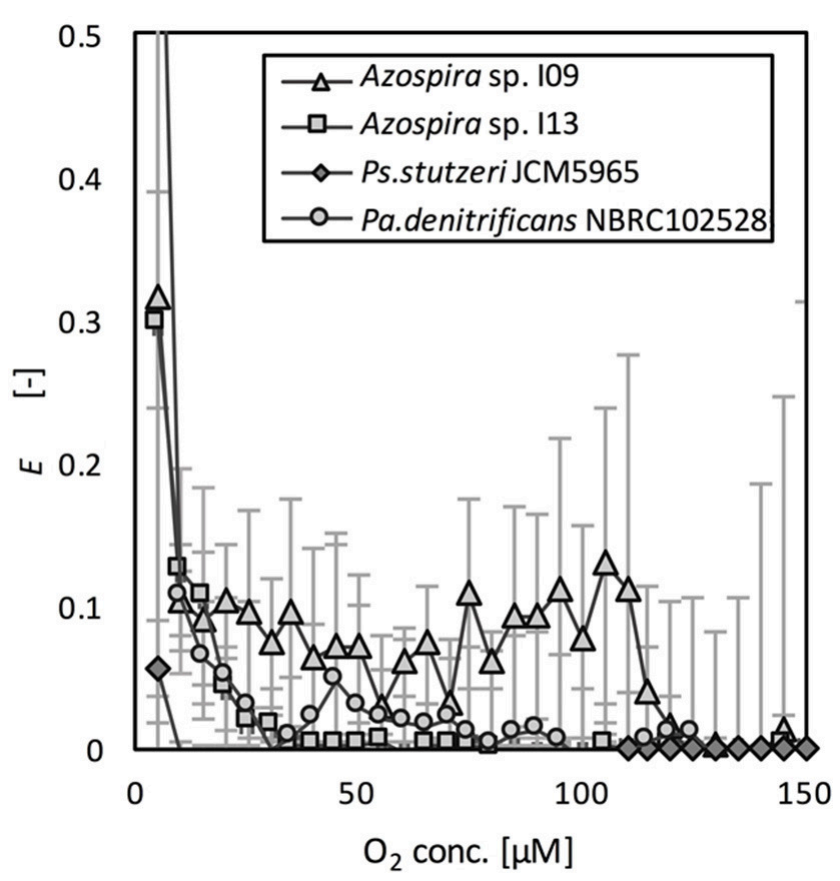
Relative N_2_O consumption rates as function of O_2_ concentration.

The effects of O_2_ on N_2_O reduction were compared on the basis of the relative activities of N_2_O reduction, *E* (Equation 4). *E* as a function of O_2_ concentration is shown in Figure 3. The effect of the DO concentration on *E* differed among the tested strains. Although the activity was lower (10% of *V*_m,N2O_) than that in the absence of oxygen, *Azospira* sp. I09 showed N_2_O consumption activity under microaerophilic conditions at DO concentrations of 100–110 μM. In contrast, the DO concentration needed to initiate N_2_O consumption by *Azospira* sp. I13 and *Pa. denitrificans* was much lower (25 μM O_2_). The N_2_O consumptions of these three strains recovered significantly after complete consumption of O_2_. *Ps. stutzeri* did not consume N_2_O until the O_2_ was completely depleted.

**Figure 4:**
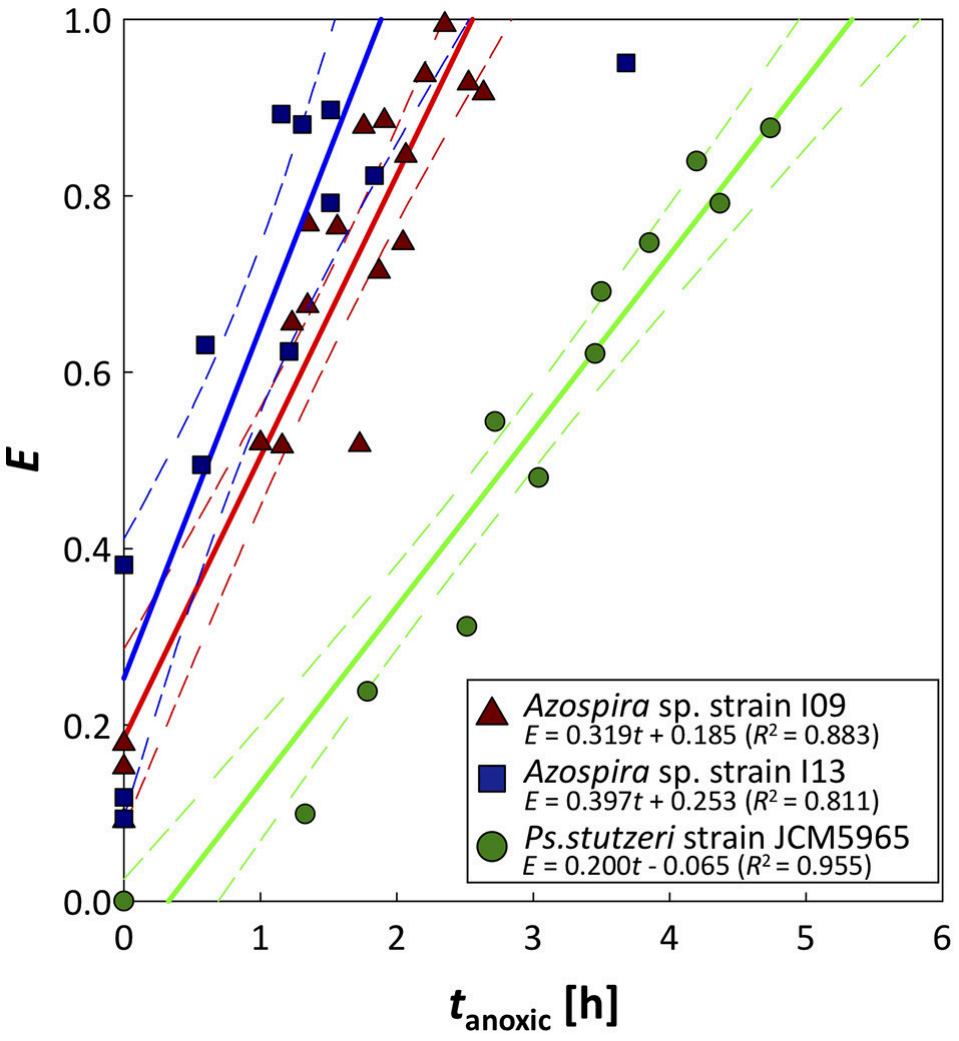
Recovery of N_2_O reduction activity after O_2_ exposure. Plots are derived from N_2_O consumption tests, performed in duplicate or triplicate: *t*_anoxic_ = 0 is elapsed time after DO is completely depleted. Solid and broken lines represent approximated activity recovery for N_2_O reduction in each strain and the 95% confidence interval of each approximated line, respectively. For the approximation, triplicate and duplicate data were used for the two *Azospira* sp. strains and *Ps. stutzeri*, respectively.

The time series for the N_2_O relative reduction rates under anoxic conditions are shown in Figure 4. *Pa. denitrificans* was excluded from the analysis because the inherently low N_2_O affinity of *Pa. denitrificans* hampered accurate measurement of the N_2_O reduction rate (Table 1). Relative activity of N_2_O reduction rate increased linearly in the three strains. The two *Azospira* sp. strains showed the same trend in *E*-values, and regained 0.8 of the initial value in 1.93 h (I09) and 1.38 h (I13), respectively. In contrast, *Ps. stutzeri* required 4.32 h for 80% recovery of *E* (Figure 4). The Nos activation rates (*V*_Nos_), i.e., the slopes in Figure 4, were estimated to be 0.319 ± 0.028, 0.397 ± 0.064, and 0.200 ± 0.013 h^−1^ for *Azospira* sp. strain I09, I13, and *Ps. stutzeri*, respectively, indicating the highest *V*_Nos_ value for *Azospira* sp. strain I13.

The effect of O_2_ on the N_2_O reduction activity was quantitatively compared by determination of biokinetic parameters (Figure S5). *K*_I,O2_ was estimated by the model fitting for N_2_O profiles (Figure S5). *Azospira* sp. strain I09 showed the highest *K*_I,O2_ value of 2.33 ± 1.7 μM while those of *Azospira* sp. strain I13 and *Ps. stutzeri* were below 0.5 μM as summarized in Table 1.

### *nosZ* gene transcription of *Azospira* sp. strain I09

**Figure 5:**
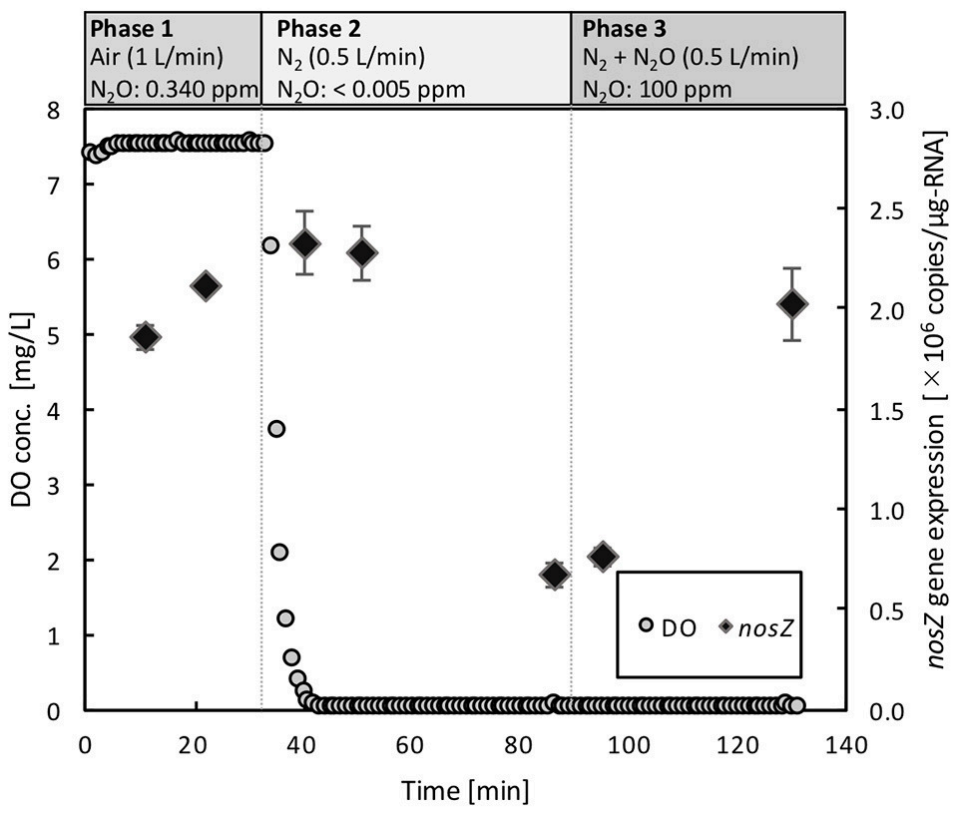
Dynamics of *nosZ* gene expression in *Azospira* sp. I09 under three different redox conditions. The gaseous compositions were ambient air (N_2_O: 0.34 ppm) in Phase 1, N_2_ (N_2_O: <0.005 ppm) in Phase 2, and N_2_ + N_2_O (N_2_O: 100 ppm) in Phase 3, respectively. The gas flow rate was consistently 0.5 L/min.

Dynamics of *nosZ* transcription of *Azospira* sp. strain I09, displaying N_2_O uptake even at above 100 μM O_2_, was monitored under different redox conditions (Figure 5). During Phase 1 for aerobic pre-incubation by air bubbling, *nosZ* transcription level slightly increased. Switching to the anoxic condition by pure N_2_ bubbling decreased the *nosZ* transcription level after 40 min by 74%. Subsequently, mixing N_2_O with N_2_ gas to keep N_2_O concentration of 100 ppmv (equivalent dissolved N_2_O of 2.16 μM at 30°C) in Phase 3 stimulated *nosZ* transcription, reaching the comparable level in Phase 1.

## Discussion

This study, for the first time, showed the effects of oxygen on the N_2_O reduction activities of *nosZ* clade I and II type N_2_O-reducing bacteria in a comparative manner. Monitoring N_2_O consumption dynamics has always been challenging because of the difficulty in setting up conditions under which the N_2_O and O_2_ concentrations are always transitioning. A common batch experiment, measuring the headspace gas in a vial (Bueno et al., 2015), tackled the difficulty of monitoring N_2_O and O_2_ concentrations within short intervals. The micro-respirometric system using DO and N_2_O microsensors used in this study enabled real-time monitoring of these dissolved gases at a much higher resolution (second level) than in conventional methods. The highly resolved profiles detect N_2_O-reducing activity even under aerobic conditions. We found that physiological characteristics do not clearly distinguish clade I and II type N_2_O-reducing bacteria.

There has been open discussion on whether clade II type N_2_O-reducing bacteria have inherently high N_2_O affinities compared with those of their clade I type counterparts (Yoon et al., 2016; Hallin et al., 2018). Current research suggested the existence of unique electron transport module in clade II type microorganisms, i.e., *Wolinella succinogenes* (Hein et al., 2017). The feature is advantageous for low-energy translocation pathways and effective energy conversion within the N_2_O respiration compared to *nosZ* clade I type bacteria. Our biokinetic analyses of the clade II type N_2_O-reducing bacterium, i.e., two *Azospira* sp. strains, show that their half-saturation constants (*K*_m,N2O_) are lower than those of the two clade I type denitrifying bacteria (Table 1). This is in agreement with previous studies, in which clade II type strains, namely *Dechloromonas aromatica* strain RCB and *Anaeromyxobacter dehalogenans* strain 2CP-C, gave lower *K*_m,N2O_ and higher biomass yields than did clade I type strains (Yoon et al., 2016). Further comparison, with the substrate affinity *a*_0,N2O_ as an indicator of N_2_O-reducing activity, shows that clade II type *Azospira* sp. strain I13 is a promising N_2_O sink (Table 1). However, even for the same *Azospira* sp., the two isolates had distinct *a*_0,N2O_ values. Furthermore, the recent report demonstrated that a chemostat system, supplying N_2_O as a sole electron acceptor, enriched clade I type N_2_O reducing bacteria with high *a*_0,N2O_ (Conthe et al., 2018). Given that taxonomic proximity is not necessarily linked to physiology, N_2_O-reducing bacteria having very high affinity to N_2_O could be broadly distributed irrespective *nosZ* clade type. Comprehensive biokinetic analysis focusing on not only clade II type but also clade I type is needed.

The N_2_O reduction activities in the presence of O_2_ differed among the four bacterial strains, irrespective of their *nosZ* clade types. *Azospira* sp. strain I09 exhibited N_2_O-reducing activity at a DO concentration of 110 μM (3.52 mg/L) and higher O_2_ inhibition coefficient (2.33 μM), confirming the participation of aerobic N_2_O reduction. Previous physiological studies using isolates or mixed cultures implied that some bacteria are capable of denitrifying and respiring N_2_O under aerobic or microaerobic conditions, but the level of DO concentration needed for detectable N_2_O-reducing activity depends on the bacterial strain (Liu et al., 2013). *Thauera* sp. strain 63 expresses *nosZ* mRNA under microaerobic conditions (<10 μM), although *T. terpenica* strain DSM12139 does so under obligate anaerobic conditions (Liu et al., 2013). The Nos of *Ps. stutzeri* strain ATCC14405 is expressed at a DO concentration of 119 μM (Körner and Zumft, 1989) and *Ps. stutzeri* strain TR2 showed N_2_O-reducing activity at a DO concentration of 35 μM (Miyahara et al., 2010). In a mixed culture, ${\text{NO}}_{2}^{-}$ reduction activity by microorganisms in soils appears at a DO concentration of 80 μM (Morley et al., 2008), and denitrification gene transcription is continuously detected under O_2_-remaining conditions in coastal sediments (Marchant et al., 2017). On the basis of previous reports, the DO level for the emergence of N_2_O consumption by *Azospira* sp. strain I09 (110 μM) is higher than those of *Thauera* sp. strain 63 and *Ps. stutzeri* strain TR2, and comparable to that of *Ps. stutzeri* strain ATCC14405. Thorough, holistic investigations are needed, but our results suggest that *Azospira* sp. strain I09 has promise as a N_2_O consumer under microaerophilic conditions.

The activity tolerance of O_2_ inhibition was reasonably described applying the Nos activation rate, *V*_Nos_ (Figure 4). The higher values of *V*_Nos_ for the *Azospira* sp. strains (0.319 ± 0.028h^−1^ for I09 and 0.397 ± 0.064 h^−1^ for I13) than that for *Ps. stutzeri* (0.200 ± 0.013 h^−1^) indicate that the recovery of N_2_O reduction activity by the *Azospira* sp. strains is faster than that by *Ps. stutzeri*. In addition, *E*-values for the *Azospira* sp. strains higher than 0 at *t*_anoxic_ = 0 suggests their superior N_2_O uptake resilience to O_2_ exposure, which was not observed for *Ps. stutzeri* (Figure 4). In previous reports, two mechanisms of O_2_ inhibition to N_2_O reduction activity have been hypothesized, namely regulation of the RNA transcription level (Arai, 2011; Bergaust et al., 2012) and Nos inactivation (Otte et al., 1996; Miyahara et al., 2010). The quantification of *nosZ* clade II type mRNA transcripts for *Azospira* sp. strain I09 under different redox conditions likely provided Nos reactivation as a limiting factor to determine N_2_O uptake rate. The application of RT-qPCR demonstrated that expression of the *nosZ* mRNA by *Azospira* sp. strain I09 was suppressed in the absence of O_2_ and N_2_O but not in the presence of N_2_O and O_2_ (Phase 1 and Phase 3 in Figure 5). This agrees with the trend that *nosZ* gene is expressed even in the presence of O_2_ in coastal sediments (Marchant et al., 2017). The consistent trend corroborates that Nos is probably synthesized even under aerobic or microaerobic conditions. Studies on extracted and purified Nos support Nos recovery after switching from aerobic to anoxic conditions (Ghosh et al., 2003; Chan et al., 2004). On the basis of these observations, it is likely that recovery of N_2_O consumption depends on the rate of Nos reactivation.

Toward N_2_O mitigation in engineered systems, the degree of Nos reactivation after O_2_ exposure should be quantitatively estimated. Combination of a respirometric analysis with a mechanistic modeling potentially accelerates selection of highly efficient N_2_O-reducing bacteria able to respire N_2_O under microaerophilic conditions. The heterotrophic denitrification model, incorporating the O_2_ inhibition (Equation 3) (Ni et al., 2011), likely necessitates the extension due to some discrepancies with the experimental results (Figure S5). Validation and verification of the extended models, e.g., an enzyme-explicit denitrification model, where a ratio of inactive Nos to total Nos and its recovery rate determine N_2_O uptake rate (Zheng and Doskey, 2015) and an integrated model incorporating the O_2_ inhibition and Nos recovery, warrant future intensive studies.

This study shows that strains of the genus *Azospira* are promising N_2_O reducers because of their O_2_ inhibition tolerance and high affinity for N_2_O. Use of the proposed kinetic parameters, *V*_Nos_ and *K*_I,O2_, provides, for the first time, quantitative information on Nos recovery from O_2_ inhibition. Application of respirometric biokinetic analysis will be useful in developing N_2_O mitigation strategies using N_2_O reducers in engineered systems. Comprehensive screening of N_2_O-reducing bacteria displaying high N_2_O affinities (*a*_0,N2O_) and high resilience against O_2_ exposure (*V*_Nos_, *K*_I,O2_) is required.

## Conclusion

This study investigated the effects of O_2_ on the N_2_O consumption biokinetics of bacterial isolates affiliated to *nosZ* clade I and II types. Respirometric assays showed that the N_2_O affinities of two *Azospira* sp. strains of *nosZ* clade II type were higher than those of *Pa. denitrificans* and *Ps. stutzeri* of *nosZ* clade I type. However, the clade type does not completely explain the different N_2_O affinities, and this suggests that the physiological traits of N_2_O-reducing bacteria differ at the species and strain levels. The N_2_O consumption activities are also significantly affected by the O_2_ levels but the degree of O_2_ inhibition differs depending on the species or strain rather than the *nosZ* type. *Azospira* sp. strain I09 showed aerobic N_2_O-reducing activity and *nosZ* gene transcription at a DO concentration of 110 μM. Recoveries of the N_2_O-reducing activities of *Azospira* sp. strains I09 and I13 after O_2_ exposure are faster than that of *Ps. stutzeri* of *nosZ* clade I type. This underscores that the Nos of *Azospira* spp. is reactivated after switching from aerobic to anoxic conditions. Our results provide new information on the physiological characteristics of N_2_O-reducing bacteria, and also a valuable investigative parameter involving a respirometric analysis. This comprehensive and thorough analyses based on a respirometric approach will accelerate the discovery of novel N_2_O reducers and contribute to N_2_O mitigation in engineering applications.

## Author contributions

AT: designed and led the study. TS: performed the biokinetic estimation and oxygen effect on nitrous oxide reduction of the isolates. TS: wrote the paper with major edits and inputs from SR, MH, and AT.

### Conflict of interest statement

The authors declare that the research was conducted in the absence of any commercial or financial relationships that could be construed as a potential conflict of interest.

## References

[B1] Ali M. Rathnayake R. M. L. D. Zhang L. Ishii S. Kindaichi T. Satoh H.. (2016). Source identification of nitrous oxide emission pathways from a single-stage nitritation-anammox granular reactor. Water Res. 102, 147–157. 10.1016/j.watres.2016.06.034 27340816

[B2] Arai H. (2011). Regulation and function of versatile aerobic and anaerobic respiratory metabolism in *Pseudomonas aeruginosa*. Front. Microbiol. 2:103. 10.3389/fmicb.2011.00103 21833336 PMC3153056

[B3] Baumann B. Snozzi M. Zehnder A. J. Van Der Meer J. R. (1996). Dynamics of denitrification activity of *Paracoccus denitrificans* in continuous culture during aerobic-anaerobic changes. J. Bacteriol. 178, 4367–4374. 10.1128/jb.178.15.4367-4374.1996 8755862 PMC178201

[B4] Bergaust L. van Spanning R. J. Frostegård Å. Bakken L. R. (2012). Expression of nitrous oxide reductase in *Paracoccus denitrificans* is regulated by oxygen and nitric oxide through FnrP and NNR. Microbiology 158, 826–834. 10.1099/mic.0.054148-0 22174385 PMC3541799

[B5] Black A. Hsu P. C. L. Hamonts K. E. Clough T. J. Condron L. M. (2016). Influence of copper on expression of *nirS, norB* and *nosZ* and the transcription and activity of NIR, NOR and N_2_OR in the denitrifying soil bacteria *Pseudomonas stutzeri*. Microb. Biotechnol. 9, 381–388. 10.1111/1751-7915.12352 26935976 PMC4835574

[B6] Bueno E. Mania D. Frostegard Å. Bedmar E. J. Bakken L. R. Delgado M. J. (2015). Anoxic growth of *Ensifer meliloti* 1021 by N_2_O-reduction, a potential mitigation strategy. Front. Microbiol. 6:537. 10.3389/fmicb.2015.00537 26074913 PMC4443521

[B7] Chan J. M. Bollinger J. A. Grewell C. L. Dooley D. M. (2004). Reductively activated nitrous oxide reductase reacts directly with substrate. J. Am. Chem. Soc. 126, 3030–3031. 10.1021/ja0398868 15012115

[B8] Conthe M. Wittorf L. Kuenen J. G. Kleerebezem R. van Loosdrecht M. C. M. Hallin S. (2018). Life on N_2_O: deciphering the ecophysiology of N_2_O respiring bacterial communities in a continuous culture. ISME J. 12, 1142–1153. 10.1038/s41396-018-0063-7 29416125 PMC5864245

[B9] Domeignoz-Horta L. A. Spor A. Bru D. Breuil M. C. Bizouard F. Léonard J.. (2015). The diversity of the N_2_O reducers matters for the N_2_O:N_2_ denitrification end-product ratio across an annual and a perennial cropping system. Front. Microbiol. 6:971. 10.3389/fmicb.2015.00971 26441904 PMC4585238

[B10] Ghosh S. Gorelsky S. I. Chen P. Cabrito I. MouraMoura I. Solomon E. I. (2003). Activation of N_2_O reduction by the fully reduced μ4-sulfide bridged tetranuclear CuZ cluster in nitrous oxide reductase. J. Am. Chem. Soc. 125, 15708–15709. 10.1021/ja038344n 14677937

[B11] Hallin S. Philippot L. Sanford R. A. Jones C. M. (2018). Genomics and ecology of novel N_2_O-reducing microorganisms. Trends Microbiol. 26, 43–55. 10.1016/j.tim.2017.07.003 28803698

[B12] Harter J. Krause H.-M. Schuettler S. Ruser R. Fromme M. Scholten T.. (2014). Linking N_2_O emissions from biochar-amended soil to the structure and function of the N-cycling microbial community. ISME J. 8, 660–674. 10.1038/ismej.2013.160 24067258 PMC3930306

[B13] Hein S. Witt S. Simon J. (2017). Clade II nitrous oxide respiration of *Wolinella succinogenes* depends on the NosG, -C1, -C2, -H electron transport module, NosB and a Rieske/cytochrome bc complex. Environ. Microbiol. 19, 4913–4925. 10.1111/1462-2920.13935 28925551

[B14] Henry S. Bru D. Stres B. Hallet S. Philippot L. (2006). Quantitative detection of the *nosZ* gene, encoding nitrous oxide reductase, and comparison of the abundances of 16S rRNA, *narG, nirK*, and *nosZ* genes in soils. Appl. Environ. Microbiol. 72, 5181–5189. 10.1128/AEM.00231-06 16885263 PMC1538733

[B15] Huang G. Z. Dong R. H. Allen R. Davis E. L. Baum T. J. Hussey R. S. (2005). Developmental expression and molecular analysis of two *Meloidogyne incognita* pectate lyase genes. Int. J. Parasitol. 35, 685–692. 10.1016/j.ijpara.2005.01.006 15862581

[B16] IPCC (2013). Climate Change 2013: The Physical Science Basis. Contribution of Working Group I to the Fifth Assessment Report of the Intergovernmental Panel on Climate Change, IPCC.

[B17] Ishii S. Song Y. Rathnayake L. Tumendelger A. Satoh H. Toyoda S.. (2014). Identification of key nitrous oxide production pathways in aerobic partial nitrifying granules. Environ. Microbiol. 16, 3168–3180. 10.1111/1462-2920.12458 24650173

[B18] Jones C. M. Graf D. R. Bru D. Philippot L. Hallin S. (2013). The unaccounted yet abundant nitrous oxide-reducing microbial community: a potential nitrous oxide sink. ISME J. 7, 417–426. 10.1038/ismej.2012.125 23151640 PMC3554408

[B19] Jones C. M. Spor A. Brennan F. P. Breuil M. C. Bru D. Lemanceau P. (2014). Recently identified microbial guild mediates soil N_2_O sink capacity. Nat. Clim. Chang. 4, 801–805. 10.1038/nclimate2301

[B20] Jones C. M. Stres B. Rosenquist M. Hallin S. (2008). Phylogenetic analysis of nitrite, nitric oxide, and nitrous oxide respiratory enzymes reveal a complex evolutionary history for denitrification. Mol. Biol. Evol. 25, 1955–1966. 10.1093/molbev/msn146 18614527

[B21] Juhanson J. Hallin S. Söderström M. Stenberg M. Jones C. M. (2017). Spatial and phyloecological analyses of nosZ genes underscore niche differentiation amongst terrestrial N_2_O reducing communities. Soil Biol. Biochem. 115, 82–91. 10.1016/j.soilbio.2017.08.013

[B22] Körner H. Frunzke K. Döhler K. Zumft W. G. (1987). Immunochemical patterns of distribution of nitrous oxide reductase and nitrite reductase (cytochrome cd1) among denitrifying pseudomonads. Arch. Microbiol. 148, 20–24. 10.1007/BF00429641 3115219

[B23] Körner H. Zumft W. G. (1989). Expression of denitrification enzymes in response to the dissolved oxygen levels and respiratory substrate in continuous culture of *Pseudomonas stutzeri*. Appl. Environ. Microbiol. 55, 1670–1676. 2764573 10.1128/aem.55.7.1670-1676.1989 PMC202933

[B24] Law Y. Ye L. Pan Y. Yuan Z. (2012). Nitrous oxide emissions from wastewater treatment processes. Philos. Trans. R. Soc. Lond. B Biol. Sci. 367, 1265–1277. 10.1098/rstb.2011.0317 22451112 PMC3306625

[B25] Liu B. Mao Y. Bergaust L. Bakken L. R. Frostegard A. (2013). Strains in the genus *Thauera* exhibit remarkably different denitrification regulatory phenotypes. Environ. Microbiol. 15, 2816–2828. 10.1111/1462-2920.12142 23663391

[B26] Lunau M. Lemke A. Walther K. Martens-Habbena W. Simon M. (2005). An improved method for counting bacteria from sediments and turbid environments by epifluorescence microscopy. Environ. Microbiol. 7, 961–968. 10.1111/j.1462-2920.2005.00767.x 15946292

[B27] Marchant H. K. Ahmerkamp S. Lavik G. Tegetmeyer H. E. Graf J. Klatt J. M.. (2017). Denitrifying community in coastal sediments performs aerobic and anaerobic respiration simultaneously. ISME J. 11, 1799–1812. 10.1038/ismej.2017.51 28463234 PMC5520038

[B28] Martens-Habbena W. Berube P. M. Urakawa H. de la Torre J. R. Stahl D. A. Torre J.. (2009). Ammonia oxidation kinetics determine niche separation of nitrifying Archaea and Bacteria. Nature 461, 976–979. 10.1038/nature08465 19794413

[B29] Miyahara M. Kim S. W. Fushinobu S. Takaki K. Yamada T. Watanabe A.. (2010). Potential of aerobic denitrification by *Pseudomonas stutzeri* TR2 to reduce nitrous oxide emissions from wastewater treatment plants. Appl. Environ. Microbiol. 76, 4619–4625. 10.1128/AEM.01983-09 20495048 PMC2901746

[B30] Morley N. Baggs E. M. Dörsch P. Bakken L. (2008). Production of NO, N_2_O and N_2_ by extracted soil bacteria, regulation by ${\text{NO}}_{2}^{-}$ and O_2_ concentrations. FEMS Microbiol. Ecol. 65, 102–112. 10.1111/j.1574-6941.2008.00495.x 18462397

[B31] Muyzer G. Dewaal E. C. Uitterlinden A. G. (1993). Profiling of complex microbial populations by denaturing gradient gel electrophoresis analysis of polymerase chain reaction-amplified genes coding for 16S rRNA. Appl. Environ. Microbiol. 59, 695–700. 7683183 10.1128/aem.59.3.695-700.1993 PMC202176

[B32] Ni B.-J. Ruscalleda M. Pellicer-Nacher C. Smets B. F. (2011). Modeling nitrous oxide production during biological nitrogen removal via nitrification and denitrification: extensions to the general ASM models. Environ. Sci. Technol. 45, 7768–7776. 10.1021/es201489n 21780759

[B33] Orellana L. H. Rodriguez-R L. M. Higgins S. Chee-Sanford J. C. Sanford R. A. Ritalahti K. M.. (2014). Detecting nitrous oxide reductase (*nosZ*) genes in soil metagenomes: method development and implications for the nitrogen cycle. MBio 5:e01193-14. 10.1128/mBio.01193-14 24895307 PMC4049103

[B34] Otte S. Grobben N. G. Robertson L. A. Jetten M. S. Kuenen J. G. (1996). Nitrous oxide production by *Alcaligenes faecalis* under transient and dynamic aerobic and anaerobic conditions. Appl. Environ. Microbiol. 62, 2421–2426. 8779582 10.1128/aem.62.7.2421-2426.1996 PMC168025

[B35] Pan Y. Ni B. J. Bond P. L. Ye L. Yuan Z. (2013). Electron competition among nitrogen oxides reduction during methanol-utilizing denitrification in wastewater treatment. Water Res. 47, 3273–3281. 10.1016/j.watres.2013.02.054 23622815

[B36] Park D. Kim H. Yoon S. (2017). Nitrous oxide reduction by an obligate aerobic bacterium *Gemmatimonas aurantiaca* strain T-27. Appl. Environ. Microbiol. 83:e00502-17. 10.1128/AEM.00502-17 28389533 PMC5452805

[B37] Pauleta S. R. Dell'Acqua S. Moura I. (2013). Nitrous oxide reductase. Coord. Chem. Rev. 257, 332–349. 10.1016/j.ccr.2012.05.026

[B38] Philippot L. (2002). Denitrifying genes in bacterial and Archaeal genomes. Biochim. Biophys. Acta 1577, 355–376. 10.1016/S0167-4781(02)00420-7 12359326

[B39] Philippot L. Andert J. Jones C. M. Bru D. Hallin S. (2011). Importance of denitrifiers lacking the genes encoding the nitrous oxide reductase for N_2_O emissions from soil. Glob. Chang. Biol. 17, 1497–1504. 10.1111/j.1365-2486.2010.02334.x

[B40] Ravishankara A. R. Daniel J. S. Portmann R. W. (2009). Nitrous oxide (N_2_O): The dominant ozone-depleting substance emitted in the 21st century. Science 326, 123–125. 10.1126/science.1176985 19713491

[B41] Read-Daily B. L. Sabba F. Pavissich J. P. Nerenberg R. (2016). Kinetics of nitrous oxide (N_2_O) formation and reduction by *Paracoccus pantotrophus*. AMB Express 6:85. 10.1186/s13568-016-0258-0 27699705 PMC5047877

[B42] Riya S. Zhou S. Watanabe Y. Sagehashi M. Terada A. Hosomi M. (2012). CH_4_ and N_2_O emissions from different varieties of forage rice (*Oryza sativa L*.) treating liquid cattle waste. Sci. Total Environ. 419, 178–186. 10.1016/j.scitotenv.2012.01.014 22289172

[B43] Sanford R. A. Wagner D. D. Wu Q. Chee-Sanford J. C. Thomas S. H. Cruz-García C.. (2012). Unexpected nondenitrifier nitrous oxide reductase gene diversity and abundance in soils. Proc. Natl. Acad. Sci. U.S.A. 109, 19709–19714. 10.1073/pnas.1211238109 23150571 PMC3511753

[B44] Soler-Jofra A. Stevens B. Hoekstra M. Picioreanu C. Sorokin D. van Loosdrecht M. C. M. (2016). Importance of abiotic hydroxylamine conversion on nitrous oxide emissions during nitritation of reject water. Chem. Eng. J. 287, 720–726. 10.1016/j.cej.2015.11.073

[B45] Tallec G. Garnier J. Billen G. Gousailles M. (2006). Nitrous oxide emissions from secondary activated sludge in nitrifying conditions of urban wastewater treatment plants: effect of oxygenation level. Water Res. 40, 2972–2980. 10.1016/j.watres.2006.05.037 16844187

[B46] Terada A. Sugawara S. Hojo K. Takeuchi Y. Riya S. Harper W. F.. (2017). Hybrid nitrous oxide production from a partial nitrifying bioreactor: hydroxylamine interactions with nitrite. Environ. Sci. Technol. 51, 2748–2756. 10.1021/acs.est.6b05521 28164698

[B47] Terada A. Sugawara S. Yamamoto T. Zhou S. Koba K. Hosomi M. (2013). Physiological characteristics of predominant ammonia-oxidizing bacteria enriched from bioreactors with different influent supply regimes. Biochem. Eng. J. 79, 153–161. 10.1016/j.bej.2013.07.012

[B48] Vollack K. U. Zumft W. G. (2001). Nitric oxide signaling and transcriptional control of denitrification genes in *Pseudomonas stutzeri*. J. Bacteriol. 183, 2516–2526. 10.1128/JB.183.8.2516-2526.2001 11274111 PMC95168

[B49] Weiss R. F. Price B. A. (1980). Nitrous-oxide solubility in water and seawater. Mar. Chem. 8, 347–359. 10.1016/0304-4203(80)90024-9

[B50] Wunderlin P. Mohn J. Joss A. Emmenegger L. (2011). Mechanisms of N_2_O production in biological wastewater treatment under nitrifying and denitrifying conditions. Water Res. 46, 1027–1037. 10.1016/j.watres.2011.11.080 22227243

[B51] Yoon S. Nissen S. Park D. Sanford R. A. Löffler F. E. (2016). Nitrous oxide reduction kinetics distinguish bacteria harboring clade I versus clade II NosZ. Appl. Environ. Microbiol. 82, 3793–3800. 10.1128/AEM.00409-16 27084012 PMC4907195

[B52] Zheng J. Doskey P. V. (2015). Modeling nitrous oxide production and reduction in soil through explicit representation of denitrification enzyme kinetics. Environ. Sci. Technol. 49, 2132–2139. 10.1021/es504513v 25588118

[B53] Zheng M. He D. Ma T. Chen Q. Liu S. Ahmad M. (2014). Reducing NO and N_2_O emission during aerobic denitrification by newly isolated *Pseudomonas stutzeri* PCN-1. Bioresour. Technol. 162, 80–88. 10.1016/j.biortech.2014.03.125 24747385

[B54] Zhu X. Burger M. Doane T. A. Horwath W. R. (2013). Ammonia oxidation pathways and nitrifier denitrification are significant sources of N_2_O and NO under low oxygen availability. Proc. Natl. Acad. Sci. U.S.A. 110, 6328–6333. 10.1073/pnas.1219993110 23576736 PMC3631630

[B55] Zumft W. G. (1997). Cell biology and molecular basis of denitrification. Microbiol. Mol. Biol. Rev. 61, 533–616. 9409151 10.1128/mmbr.61.4.533-616.1997 PMC232623

[B56] Zumft W. G. Kroneck P. M. (2006). Respiratory transformation of nitrous oxide (N_2_O) to dinitrogen by Bacteria and Archaea. Adv. Microb. Physiol. 52, 107–227. 10.1016/S0065-2911(06)52003-X 17027372

