# Supplementary material for: Biokinetic Characterization and Activities of N2O-Reducing Bacteria in Response to Various Oxygen Levels
Source: Front Microbiol. 2018 Apr 10;9:697. doi: 10.3389/fmicb.2018.00697 (PMC5902568; doi:10.3389/fmicb.2018.00697)
Supplement: Supplementary file 1 [file Presentation1.PDF]

SUPPLEMENTARY INFORMATION

FIGURE LEGENDS

Figure S1 N_2O and O_2 respiration profiles of *Azospira* sp. strain I09; black line: O_2, gray line: N_2O. Two panels show profiles in duplicate.

Figure S2 N_2O and O_2 respiration profiles of *Azospira* sp. strain I13; black line: O_2, gray line: N_2O. Two panels show profiles in duplicate.

Figure S3 N_2O and O_2 respiration profiles of *Pseudomonas stutzeri* JCM5965; black line: O_2, gray line: N_2O. Two panels show profiles in duplicate.

Figure S4 N_2O and O_2 respiration profiles of *Paraccoccus denitrificans* strain NBRC102528; black line: O_2, gray line: N_2O. Two panels show profiles in duplicate.

Figure S5 Model fitting results for $K_{I,O2}$ estimation. For calculation, $V_{m,N2O}$ was used a first spiked value in each run.

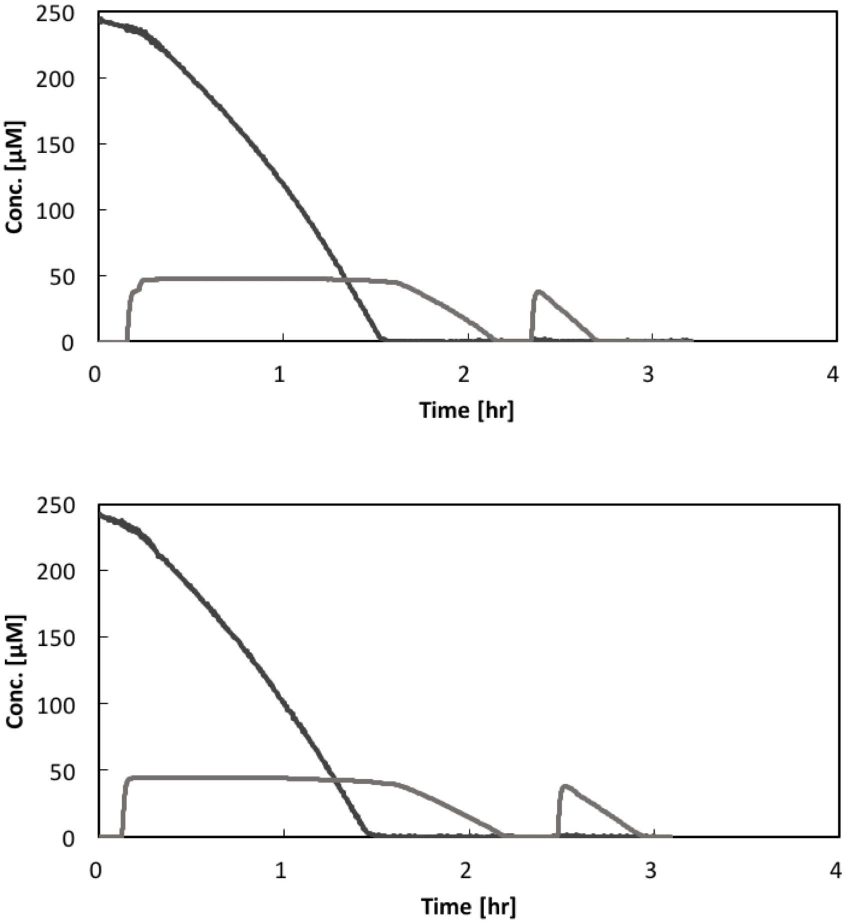
250
200
150
100
50
0
Conc. [μM]
0
1
2
3
4
Time [hr]
250
200
150
100
50
0
Conc. [μM]
0
1
2
3
4
Time [hr]

Figure S1

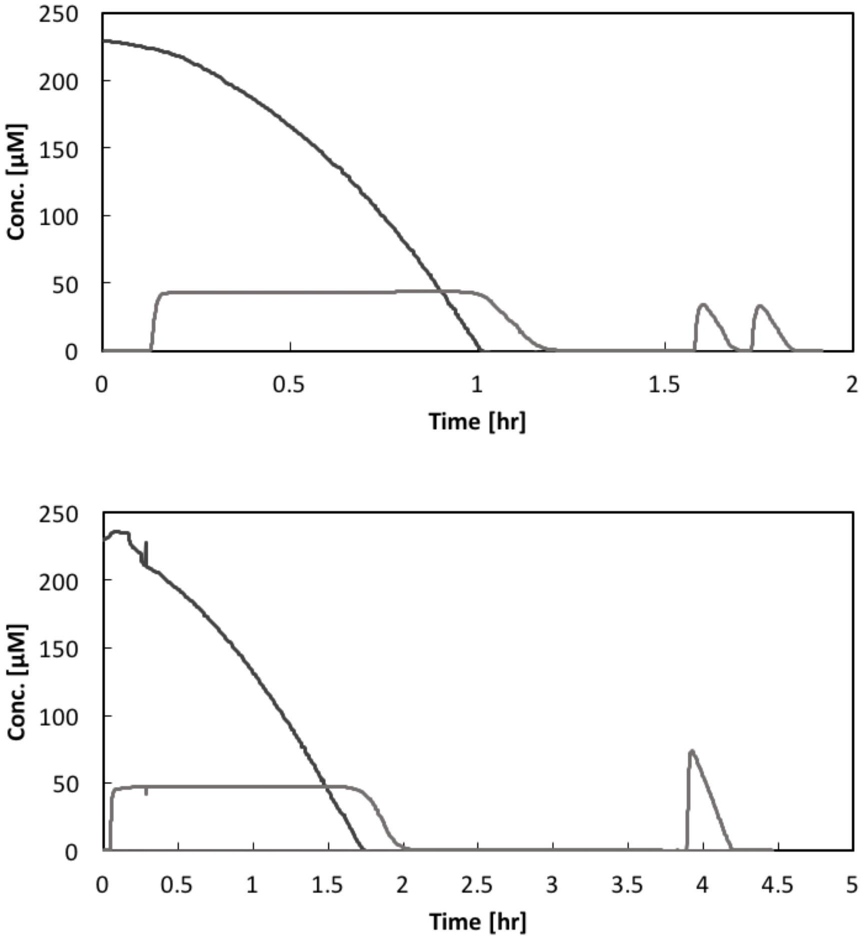
250
200
150
100
50
0
Conc. [µM]
0
0.5
1
1.5
2
Time [hr]
250
200
150
100
50
0
Conc. [µM]
0
0.5
1
1.5
2
2.5
3
3.5
4
4.5
5
Time [hr]

Figure S2

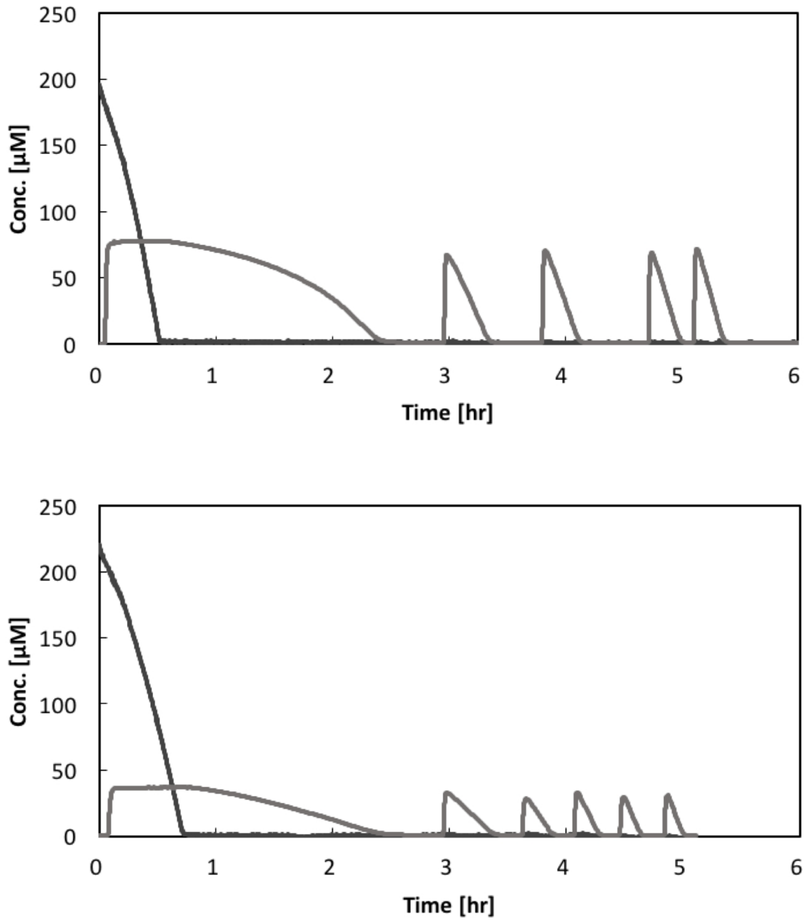
250
200
150
100
50
0
Conc. [μM]
0
1
2
3
4
5
6
Time [hr]
250
200
150
100
50
0
Conc. [μM]
0
1
2
3
4
5
6
Time [hr]

Figure S3

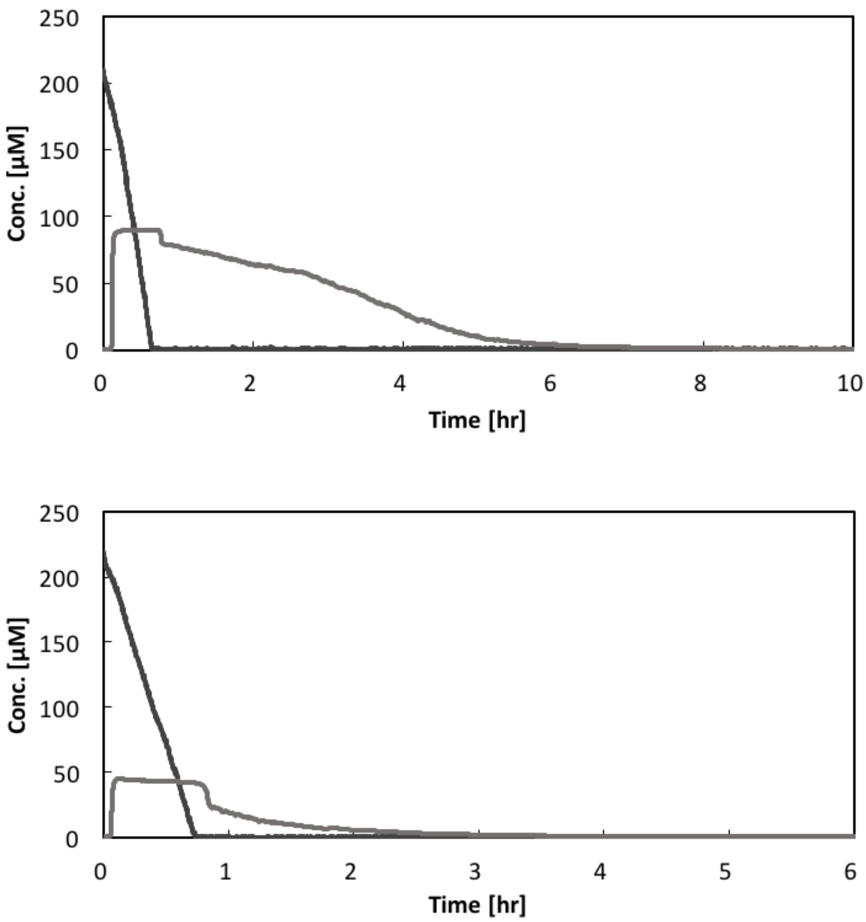
250
200
150
100
50
0
Conc. [µM]
0
2
4
6
8
10
Time [hr]
250
200
150
100
50
0
Conc. [µM]
0
1
2
3
4
5
6
Time [hr]

Figure S4

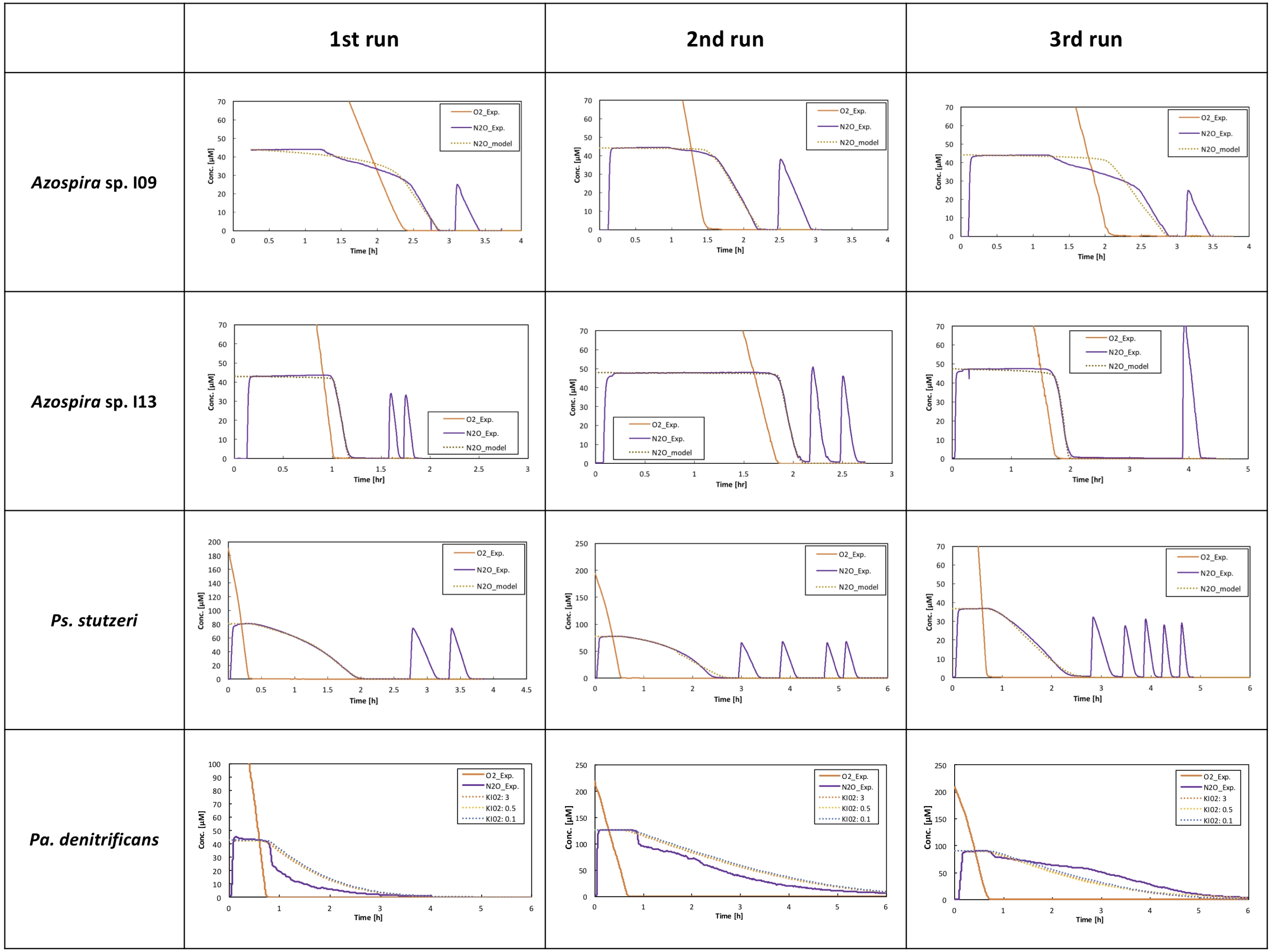
1st run
2nd run
3rd run
Azospira sp. I09
Azospira sp. I13
Ps. stutzeri
Pa. denitrificans
O2_Exp.
N2O_Exp.
N2O_model
KI02: 3
KI02: 0.5
KI02: 0.1
Conc. [µM]
Time [h]
Time [hr]

Figure S5
